# Results of a randomised Phase II trial of olaparib, chemotherapy or olaparib and cediranib in patients with platinum-resistant ovarian cancer

**DOI:** 10.1038/s41416-023-02567-6

**Published:** 2024-01-20

**Authors:** Shibani Nicum, Naomi McGregor, Rachel Austin, Linda Collins, Susan Dutton, Iain McNeish, Rosalind Glasspool, Marcia Hall, Rene Roux, Agnieszka Michael, Andrew Clamp, Gordon Jayson, Rebecca Kristeleit, Susana Banerjee, Anita Mansouri

**Affiliations:** 1 University College London Cancer Institute, London, UK; 2 Newcastle Clinical Trials Unit, Newcastle University, UK and Oncology Clinical Trials Office (OCTO), Department of Oncology, University of Oxford, Oxford, UK; 3 Oncology Clinical Trials Office (OCTO), Department of Oncology, University of Oxford, Oxford, UK; 4 Centre for Statistics in Medicine, Nuffield Department of Orthopaedics, Rheumatology and Musculoskeletal Sciences, University of Oxford, Oxford, UK; 5 Ovarian Cancer Action Research Centre Department of Surgery and Cancer, Imperial College London, London, UK; 6 Beatson West of Scotland Cancer Centre, NHS Greater Glasgow and Clyde and University of Glasgow, Glasgow, UK; 7 Department of Medical Oncology, Mount Vernon Cancer Centre, Middlesex, Northwood, UK; 8 Department of Oncology, Oxford University Hospitals NHS Foundation Trust, Oxford, UK; 9 Department of Clinical and Experimental Medicine, University of Surrey, Surrey, UK; 10 The Christie NHS Foundation Trust and Institute of Cancer Sciences, University of Manchester, Manchester, UK; 11 Guy’s and St Thomas NHS Foundation Trust and King’s College London, London, UK; 12 The Royal Marsden NHS Foundation Trust and Institute of Cancer Research, London, UK

**Keywords:** Ovarian cancer, Ovarian cancer

## Abstract

**Background:**

OCTOVA compared the efficacy of olaparib (O) versus weekly paclitaxel (wP) or olaparib + cediranib (O + C) in recurrent ovarian cancer (OC).

**Aims:**

The main aim of the OCTOVA trial was to determine the progression-free survival (PFS) of olaparib (O) versus the oral combination of olaparib plus cediranib (O + C) and weekly paclitaxel (wP) in recurrent ovarian cancer (OC).

**Methods:**

In total, 139 participants who had relapsed within 12 months of platinum therapy were randomised to O (300 mg twice daily), wP (80 mg/m^2^ d1,8,15, q28) or O + C (300 mg twice daily/20 mg daily, respectively). The primary endpoint was progression-free survival (PFS) of olaparib (O) versus olaparib plus cediranib (O + C) or weekly paclitaxel (wP). The sample size was calculated to observe a PFS hazard ratio (HR) 0.64 in favour of O + C compared to O (20% one-sided type I error, 80% power).

**Results:**

The majority had platinum-resistant disease (90%), 22% prior PARPi, 34% prior anti-angiogenic therapy, 30% germline BRCA1/2 mutations. The PFS was increased for O + C vs O (O + C 5.4 mo (2.3, 9.6): O 3.7 mo (1.8, 7.6) HR = 0.73; 60% CI: 0.59, 0.89; *P* = 0.1) and no different between wP and O (wP 3.9 m (1.9, 9.1); O 3.7 mo (1.8, 7.6) HR = 0.89, 60% CI: 0.72, 1.09; *P* = 0.69). The main treatment-related adverse events included manageable diarrhoea (4% Grade 3) and hypertension (4% Grade 3) in the O + C arm.

**Discussion:**

OCTOVA demonstrated the activity of O + C in women with recurrent disease, offering a potential non-chemotherapy option.

**Trial registration:**

ISRCTN14784018, registered on 19th January 2018 http://www.isrctn.com/ISRCTN14784018.

## Introduction

Epithelial ovarian cancer is the deadliest gynaecological cancer, with around 310,000 annual cases and 210,000 deaths worldwide (Globocan 2020 https://gco.iarc.fr/today/data/factsheets/cancers/25-Ovary-fact-sheet.pdf). The majority of women present with advanced disease and, whilst most initially respond to treatment, the large majority ultimately relapse. New treatment options that are more effective with reduced toxicity are urgently needed.

PARP inhibitors such as olaparib have revolutionised the treatment of first-line [1–3] and platinum-sensitive relapsed ovarian cancer [4–6], particularly in women who have *BRCA1/2* mutations or evidence of homologous recombination deficient (HRD) tumours. In women who have the platinum-resistant disease, trials have demonstrated response rates of around 30% [7] with olaparib compared to the 3–13% response rates seen in the non-*BRCA1/2*-mutated population/non HRD (*BRCA1/2* wild-type) population [8–10].

In an effort to improve outcomes, novel PARP inhibitor combinations have been evaluated. The addition of anti-angiogenic agents such as cediranib is thought to have a synergistic effect due to the downregulation of genes involved in Homologous Recombination (HR) inducing a functional BRCA-like environment that enhances the efficacy of PARPi [11].

This has been shown to be partially due to cediranib-induced hypoxia suppressing the expression of BRCA1/2 and RAD51 recombinase but also as a result of a direct effect on HR DNA repair through platelet-derived growth factor receptor inhibition, activation of protein phosphatase 2A and also E2F transcription factor 4/RB transcriptional corepressor like 2.

The combination of olaparib and cediranib has been shown to significantly extend PFS compared to olaparib alone in a population of women with platinum-sensitive relapsed OC (16.5 versus 8.2 months, hazard ratio HR 0.50; *P* = 0.007). The impact of this combination was most marked in the *BRCA1/2* wild-type patients: PFS (23.7 versus 5.7 months, *P* = 0.002) and overall survival (37.8 versus 23.0 months, *P* = 0.047), supporting the notion that that the addition of anti-angiogenic agents extends the benefit of PARPi in this population [12]. The activity of combination olaparib and cediranib was subsequently confirmed in a Phase III trial, although this combination was not superior to carboplatin-based chemotherapy [13].

The OCTOVA trial was designed to evaluate the activity and toxicity of olaparib and the combination of olaparib and cediranib as a tolerable oral therapy in place of further intravenous chemotherapy, in women with recurrent ovarian cancer. For women who have multiply relapsed cancer, the option of a tolerable, oral therapy rather than weekly intravenous treatment would be an important advance in terms of quality of life.

## Methods

### Study design and participants

OCTOVA was an academic, randomised Phase II trial, sponsored by The University of Oxford, run in 15 centres of the UK. The protocol is available in the supplement and has been published [14].

Eligible participants had ovarian, fallopian tube, primary peritoneal cancer that had relapsed within 12 months of previous platinum-based therapy and had disease progression with measurable lesions according to Response Evaluation Criteria in Solid Tumours (RECIST) version v1.1 and Eastern Cooperative Oncology Group performance status ≤2. Patients could have received prior PARP inhibitor, providing that there was >6 month interval since treatment. Patients could also have received prior anti-angiogenic therapy providing that there was a > 6 month interval, except for bevacizumab where only a 6 week interval was required. Patients could not have received prior weekly paclitaxel chemotherapy for relapsed disease. Initially, the population only included participants with a germline or somatic *BRCA1/2* mutation who had relapsed within 6 months of prior platinum therapy (platinum-resistant disease). Participants with a *BRCA1/2* mutation were randomised with two stratification factors: prior PARP inhibitor exposure and prior anti-angiogenic therapy (randomisation version 1.0). However, due to the slow rate of recruitment as PARP inhibitors became part of standard practice in earlier lines of treatment, it was decided to widen the inclusion criteria to include *BRCA1/2* wild-type and *BRCA1/2* unknown participants relapsing within 12 months of prior platinum therapy (i.e., platinum-resistant and partially sensitive). *BRCA1/2* status was included as an extra stratification factor. The remaining participants were randomised to receive one of three arms with three stratification factors for prior PARP inhibitor exposure, prior anti-angiogenic therapy and *BRCA1/2* mutation status (randomisation version 2.0).

### Procedures

Eligible patients with relapsed ovarian, fallopian tube or primary peritoneal cancer were enrolled by investigators and randomly assigned in a 1:1:1 ratio to receive O (300 mg twice daily), wP (80 mg/m^2^ days 1,8,15 on a 28-day schedule) or O (300 mg twice daily) in combination with C (20 mg once daily). The starting dose of paclitaxel was 80 mg/m^2^ and dose reductions to 70 mg/m^2^ (dose level—1) and then to 60 mg/m^2^ (dose level—2) were permitted. The starting dose of O was 300 mg twice daily and dose reductions to 250 mg twice daily (dose level—1), 200 mg twice daily (dose level—2) and then to 150 mg twice daily (dose level—3) were allowed if necessary. The starting dose of C was 20 mg once daily with dose reductions to 20 mg on a 5 days on and 2 days off (intermittent schedule: dose level—1) and then to 15 mg once daily (continuous schedule: dose level—2) as there are data to suggest that intermittent scheduling allows the dose to be maintained and reduces toxicity (*AstraZeneca pers communication*). This was an open-label randomised trial.

Treatment continued until disease progression or unacceptable toxicity. Patients allocated to wP were permitted to receive olaparib following RECIST-confirmed disease progression.

Tumours were assessed every 8 (year 1) to 12 (year 2 onwards) weeks by computed tomography according to RECIST version 1.1. Adverse events were graded according to the Common Terminology Criteria for Adverse Events version 4. Quality of life outcomes based on EQ5D and EORTC-QLQ C30 and OV28 were performed at baseline and 4 weekly on day 1 of each treatment cycle and on progression.

### Randomisation

Stratified randomisation was performed centrally using a web-based system. The statistician generated the randomisation codes using block sizes of 3 and 6, with equality of distribution within each strata: prior Parp (yes/no) and prior anti-angiogenic therapy (yes/no). After 27 participants had been randomised, the inclusion of an additional stratification factor: BRCA status (No/Yes BRCA mutation). The randomisation system was updated to utilise a minimisation algorithm which included the first 27 participants to seed the algorithm and included a probabilistic element to prevent predictability of allocation.

### Outcomes

The primary endpoint was progression-free survival (PFS). Two pairs of comparisons, paclitaxel vs olaparib and olaparib plus cediranib vs olaparib were made.

Secondary endpoints were, objective response rate (according to RECIST version 1.1 and Gynecologic Cancer InterGroup (GCIG) CA125 criteria), overall survival, safety and tolerability (adverse events) of the combination of O + C and quality of life.

### Statistical analysis

A sample size of 138 (46 per arm) was required to observe a PFS HR 0.64 in favour of O + C compared to O alone and 1.44 for wP compared to O (20% one-sided type I error, 80% power, 15% dropout, one-sided *P* value < 0.2). All analyses were carried out using validated statistical software, Stata version 16.0. Two pairs of treatment comparisons were undertaken: wP vs O and O + C vs O.

The principal (main) primary outcome analysis was based on the available and evaluable cases subset of the ITT population. Participants were analysed according to the group they were originally assigned, regardless of what treatment (if any) they received. Primary outcome, progression-free survival (PFS), was evaluable if there was at least baseline and one follow-up scan (RECIST definition). While a participant was being followed for progression, an event was observable. An event was either progression according to RECIST or death. Participants were censored to the point that they were no longer followed up for progression.

A number of supporting analyses of the primary endpoint were undertaken to comply with CONSORT reporting guidance. A supporting analysis to assess the impact of non-RECIST progression (e.g., clinical progression) on the primary endpoint was performed. This analysis used any available data showing progression. Clinical progressions that occurred after the date that patients stopped being followed for progression were not included. The primary analysis was repeated for a per-protocol population that includes participants who received only their allocated intervention and in whom the dose intensity was at least 70%. This population excluded participants who did not receive any intervention. Those ineligible participants (i.e., did not have measurable disease at baseline) were excluded from the per-protocol population. Participants with non-RECIST-defined progression were included in the per-protocol population.

An extra sensitivity analysis which excludes those participants who have been taken off treatment due to shielding were performed to assess the effect of COVID-19 pandemic on primary endpoint analysis.

Event-related outcomes (PFS and OS) were analysed using Cox proportional hazard models adjusted for randomisation stratification factors and were presented using Kaplan–Meier plots. The absolute risk difference and odds ratio of objective response rates were conducted for ORR outcomes (RECIST and CA125) using logistic regression model, adjusted for stratification factors. Descriptive statistics are based on all available data. A one-sided *P* value of <0.2 was considered significant for the primary endpoint.

## Results

**Fig. 1:**
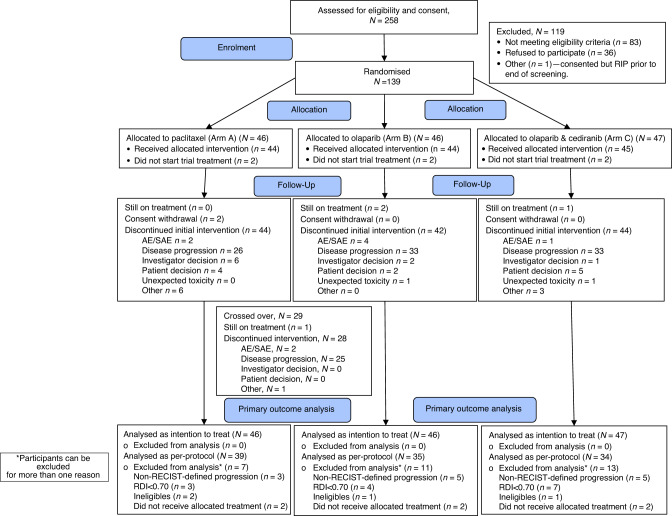
Consort diagram. AE adverse events, SAE serious adverse events.

Between 30 May 2017 and 10 January 2020, 258 patients were screened, and 139 eligible participants, median 66 years (IQR: 57–72), were enrolled from 15 sites in the United Kingdom (Fig. 1). In total, 46 patients were allocated to Arm A, wP, 46 patients to Arm B, O and 47 patients to Arm C, O + C. Two patients in each Arm did not start study treatment; the remainder (44, 44 and 45 in wP, O and O + C, respectively) received the allocated intervention. Four participants (one after cross-over) were still on treatment (as specified in the protocol) (Fig. 1). Baseline demographics and tumour characteristics are shown in Table 1. Overall 31 patients (22%) had received prior PARPi therapy; 47 (34%) prior anti-angiogenic therapy; 42 (30%) had known germline *BRCA1/2* mutations; 125 (90%) had relapsed <6 months after prior platinum. The median number of prior lines of chemotherapy was 2 (range 1–7).

**Table 1:** Baseline patient and tumour characteristics.

	Arm A: paclitaxel only (*n* = 46)	Arm B: olaparib only (*n* = 46)	Arm C: olaparib and cediranib (*n* = 47)	Total (*n* = 139)
Stratification factor PARP*				
No	36 (78%)	36 (78%)	36 (77%)	108 (78%)
Yes	10 (22%)	10 (22%)	11 (23%)	31 (22%)
Stratification factor angiogenic				
No	31 (67%)	31 (67%)	30 (64%)	92 (66%)
Yes	15 (33%)	15 (33%)	17 (36%)	47 (34%)
Stratification factor BRCA**				
No	35 (76%)	31 (67%)	31 (66%)	97 (70%)
Yes	11 (24%)	15 (33%)	16 (34%)	42 (30%)
Age at randomisation (years)				
* N* (%), missing	46 (100%), 0	46 (100%), 0	47 (100%), 0	139 (100%), 0
Mean (SD)	64 (11)	64 (10)	64 (10)	64 (10)
Median (IQR)	65 (56, 72)	65 (58, 72)	66 (56, 72)	66 (57, 72)
Min–Max	41–82	39–81	42–84	39–84
Ethnicity				
White British	38 (83%)	32 (70%)	41 (87%)	111 (80%)
White and Black African	–	1 (2%)	–	1 (1%)
Irish	–	1 (2%)	–	1 (1%)
Indian	–	–	2 (4%)	2 (1%)
Other White background	7 (15%)	10 (22%)	3 (6%)	20 (14%)
Other Asian background	–	2 (4%)	–	2 (1%)
Other mixed background	1 (2%)	–	–	1 (1%)
Other ethnic groups	–	–	1 (2%)	1 (1%)
ECOG performance status				
0	16 (35%)	9 (20%)	18 (38%)	43 (31%)
1	29 (63%)	35 (76%)	25 (53%)	89 (64%)
2	1 (2%)	2 (4%)	4 (9%)	7 (5%)
Histology				
Carcinosarcoma	1 (2%)	1 (2%)	1 (2%)	3 (2%)
Clear cell	1 (2%)	1 (2%)	–	2 (1%)
Endometrioid	1 (2%)	1 (2%)	–	2 (1%)
Serous tumour	43 (93%)	43 (93%)	46 (98%)	132 (95%)
Germline BRCA status				
BRCA test not done	4 (9%)	1 (2%)	4 (9%)	9 (6%)
* BRCA1* mutation	9 (20%)	9 (20%)	11 (23%)	29 (21%)
* BRCA2* mutation	2 (4%)	6 (13%)	5 (11%)	13 (9%)
Variant of unknown significance	1 (2%)	3 (7%)	2 (4%)	6 (4%)
Wild-type	30 (65%)	27 (59%)	25 (53%)	82 (59%)
Platinum-free interval ***				
0–3 months	27 (59%)	21 (46%)	29 (62%)	77 (55%)
3–6 months	16 (35%)	18 (39%)	14 (30%)	48 (35%)
6–12 months	3 (7%)	7 (15%)	4 (9%)	14 (10%)
Number of prior lines of chemotherapy				
1	9 (20%)	6 (13%)	8 (17%)	23 (17%)
2	17 (37%)	22 (48%)	18 (38%)	57 (41%)
3	11 (24%)	9 (20%)	11 (23%)	31 (22%)
4	6 (13%)	5 (11%)	8 (17%)	19 (14%)
5	1 (2%)	2 (4%)	2 (4%)	5 (4%)
6	2 (4%)	1 (2%)	–	3 (2%)
7	–	1 (2%)	–	1 (1%)
Number of prior lines of platinum-based chemotherapy				
1	14 (30%)	8 (17%)	10 (21%)	32 (23%)
2	15 (33%)	20 (43%)	16 (34%)	51 (37%)
3	9 (20%)	11 (24%)	14 (30%)	34 (24%)
4	6 (13%)	3 (7%)	6 (13%)	15 (11%)
5	1 (2%)	3 (7%)	1 (2%)	5 (4%)
6	1 (2%)	1 (2%)	–	2 (1%)

*Two participants were incorrectly randomised by sites, correct status is reported.

**Three participants were judged as BRCA wild-type or unknown at the beginning of the study but were later confirmed as BRCA mutant, one participant was incorrectly randomised by sites, correct status is reported.

***Date from previous platinum-based therapy to progression (therapies received before start of trial).

### Treatment compliance/dose intensity

The median (IQR) number of cycles was Arm A, wP: 4 (2, 6); Arm B, O: 4 (2, 7); Arm C, O + C: 5 (2, 7). The median dose intensity, defined as the amount of drug delivered/planned per unit of time, for wP was 0.98 (IQR 0.88, 1.00), for O 0.93 (IQR 0.74, 0.99) and for O + C 0.92 (IQR 0.79, 0.99). The median (IQR) duration of therapy in days was wP: 95.5 (43, 155); O: 109 (56, 200.5); O + C: olaparib 140 (56, 224), cediranib 140 (56, 196).

### Primary endpoint—progression-free survival

**Fig. 2:**
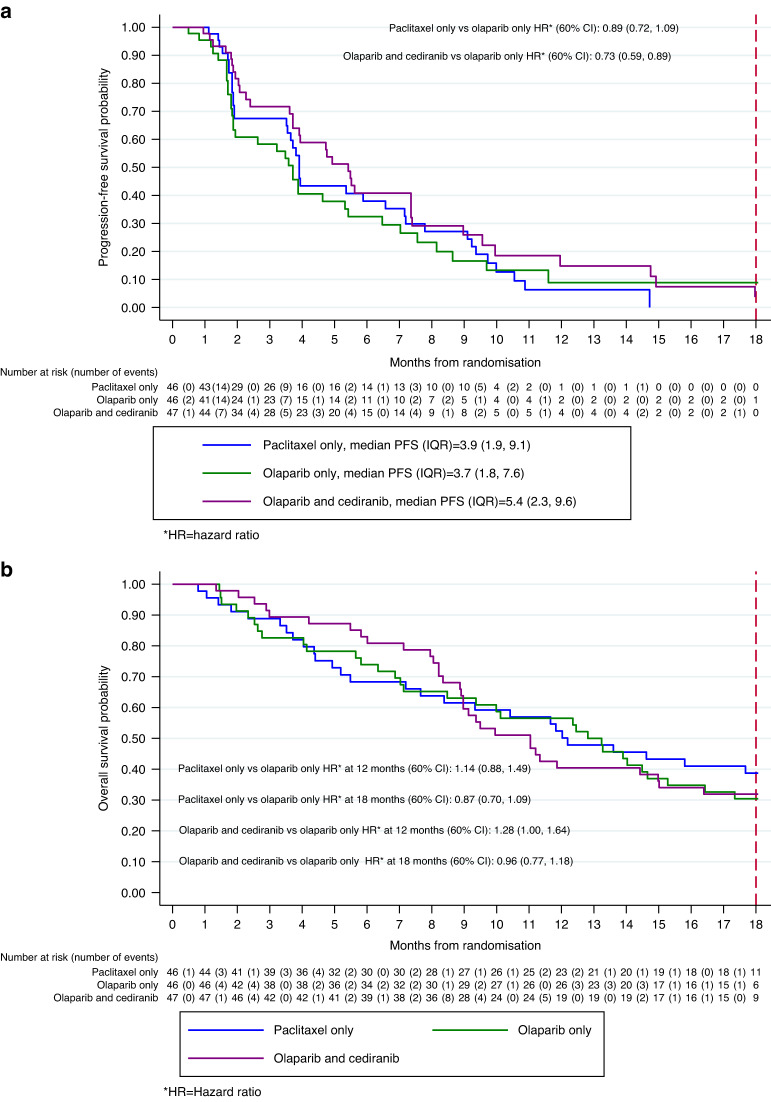
Kaplan-Meier graphs of progression free survival (a) and overall survival (b) for the intention to treat population. **a** Kaplan–Meier display of progression-free survival for the ITT analysis. **b** Kaplan–Meier display of overall survival for the ITT analysis.

With a median follow-up of 18 months, disease progression or death occurred in 106/139 (76%) participants. The Kaplan–Meier (KM) estimates for median (IQR) progression-free survival in the ITT population were wP: 3.9 m (1.9, 9.1), O: 3.7 m (1.8, 7.6) and O + C: 5.4 (2.3, 9.6) (Fig. 2a). The Cox proportional hazards model showed no significant difference in PFS between wP and O (HR = 0.89, 60% CI: 0.72, 1.09; *P* = 0.69). However, PFS was increased for O + C versus O (HR = 0.73; 60% CI: 0.59, 0.89; *P* = 0.1).

The primary analysis was repeated for a per-protocol population limited to only eligible participants who received their allocated intervention and in whom the dose intensity was at least 70% and excluded those with non-RECIST-defined progression. Results were similar to the main ITT analysis with the Cox model demonstrating no difference in PFS between wP and O (HR = 0.77, 60% CI: 0.62, 0.95) and a statistically significant increase in PFS for O + C versus O (HR = 0.61; 60% CI: 0.49, 0.77).

A supporting analysis to assess the impact of non-RECIST progression (e.g. clinical progression) on the primary endpoint was also performed. This analysis used any available data showing progression but excluded clinical progressions that occurred after the date that patients stopped being followed for progression. The finding of this analysis supported the main ITT analysis: wP versus O (HR = 0.86, 60% CI: 0.71, 1.05); O + C versus O (HR = 0.71; 60% CI: 0.59, 0.87).

### Secondary endpoints

#### Objective response rates

The proportion of patients with a confirmed partial response as the maximum response by RECIST v1.1 was 15/44 (34%) for paclitaxel; 6/43 (14%) partial response, and 1/43 (2%) complete response for O and 6/44 (14%) for O + C. The proportion of patients with a confirmed partial response or stable disease as the maximum RECIST response was 29/44 (66%) for paclitaxel vs 26/43 (60%) for O and 31/44 (70%) for O + C. The objective response rates by CA125 GCIG criteria were 47% wP vs 19% O and 31% O + C (Table 2).

**Table 2:** Objective response rates by RECIST v1.1 and CA125 by GCIG criteria.

	Paclitaxel only (*n* = 46)	Olaparib only (*n* = 46)	Olaparib and cediranib (*n* = 47)
RECIST best response rate			
Complete response	0 (0%)	1 (2%)	0 (0%)
Partial response	15 (33%)	6 (13%)	6 (13%)
Stable disease	14 (30%)	19 (41%)	25 (56%)
Progressive disease	13 (28%)	14 (30%)	11 (23%)
Non-evaluable at baseline	2 (4%)	3 (7%)	3 (6%)
Missing	2 (4%)	3 (7%)	2 (4%)
RECIST ORR^†^ (complete, partial stable)	29/44 (66%)	26/43 (60%)	31/44 (70%)
CA125 GCIC criteria	18/38 (47%)	6/32 (19%)	11/35 (31%)

^†^Only patients with evaluable RECIST at baseline.

#### Overall survival

At 12 and 18 months, there was no difference in overall survival (OS) for either of the two comparisons (O vs wP or O vs O + C), but the study was not powered to detect a difference (Fig. 2b). For analysis of overall survival, patients were censored at cross-over from wP to O. The proportion alive at 12 and 18 months, including those who cross-over is presented in Table 3.

**Table 3:** Proportion of patients alive at 12 and 18 months (patients on weekly paclitaxel censored at cross-over).

	Arm A: paclitaxel only proportion still alive (60% CI)	Arm B: olaparib only proportion still alive (60% CI)	Arm C: olaparib and cediranib proportion still alive (60% CI)
12-month	0.54 (0.47, 0.61)	0.57 (0.49, 0.64)	0.40 (0.34, 0.48)
18-month	0.41 (0.34, 0.49)	0.30 (0.24, 0.38)	0.32 (0.26, 0.39)
12-month (cross-over patients)	0.66 (0.56, 0.74)	–	–
18-month (cross-over patients)	0.55 (0.46, 0.64)	–	–

Proportion still alive (patients on wP censored at cross-over).

### Adverse events

**Fig. 3:**
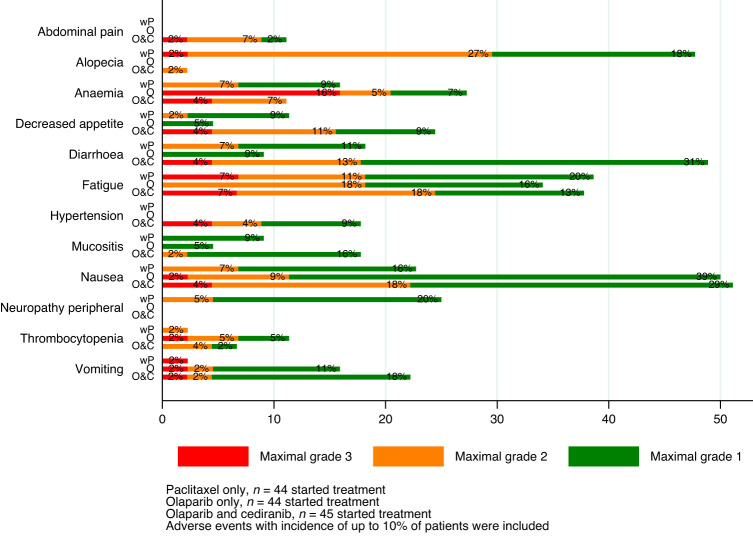
Drug related adverse events. Adverse reactions that occurred in at least 10% of patients (maximal grade).

Toxicity was more common in the combination O + C arm, but the events were predominantly grade 1 and 2 in severity and in line with the expected side effects of these agents. As expected, alopecia and neuropathy were related to treatment with wP. Anaemia was most common in the single-agent O arm, with 16% of events grade 3 compared to 4% grade 3 events in the O + C arm and mostly grade 1 and 2 anaemia in the wP arm (15%). Grade 1 and 2 nausea was prominent in both olaparib-containing arms but remained manageable. Approximately 50% patients receiving combination olaparib and cediranib experienced more events of diarrhoea, although the majority of events (33%) were grade 1 or 2 in severity. Grade 1 and 2 cediranib-associated hypertension occurred with expected incidence in O + C but was easily manageable. The incidence of fatigue was comparable across all three arms. Adverse events only led to treatment discontinuation in 4%, 9% and 2% in wP, O and O + C, respectively. Dose modification was more common in O (33%) and O + C (43%) compared to wP (11%) (Fig. 3).

### Quality of life

Quality of life outcomes based on EQ5D and EORTC-QLQ C30 and OV28 were performed at baseline and at 4 weekly intervals. No differences were observed between the three treatment arms in terms of the global health scores, so subscale comparisons have not been reported.

### Subgroup analysis

**Fig. 4:**
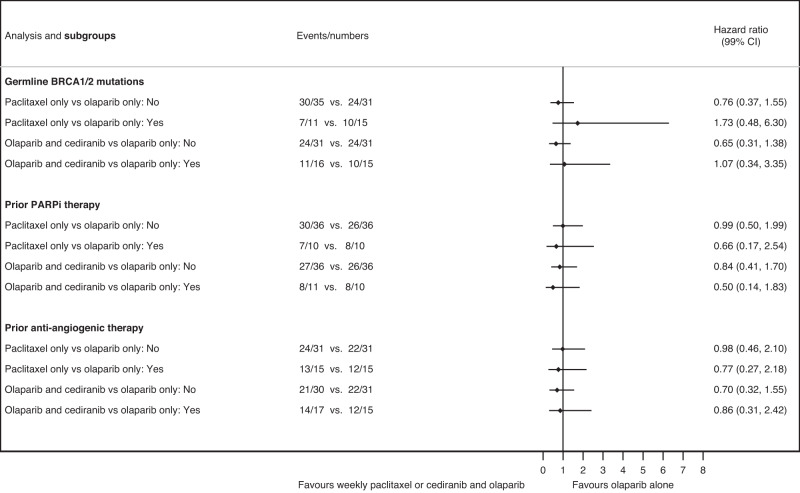
Forest plot demonstrating PFS in subgroup analysis of stratification factors.

In the ITT population, planned exploratory subgroup analyses by the three stratification factors demonstrated that the observed treatment effects in PFS across the three arms were consistent across the subgroups: prior exposure to PARPi/anti-angiogenic agents or by BRCA mutational status (confidence intervals/no *P* values as exploratory—Fig. 4). However there appeared to be a trend favouring olaparib over weekly paclitaxel for the *BRCA1/*2-mutated group although the CI is wide and crosses 1 (CI 0.48–6.3).

### COVID-19-sensitivity analysis

An extra sensitivity analysis that excluded four participants who stopped treatment due to shielding was performed to assess the effect of COVID-19 pandemic on primary endpoint analysis. To date, no patients have developed COVID whilst on study. The results from the COVID-19 sensitivity analysis were in line with and confirm the main analysis.

## Discussion

Women with multiply relapsed platinum-resistant disease have limited treatment options. In this setting, patients prioritise the effective palliation of symptoms whilst minimising treatment-related toxicity, such as alopecia and neuropathy, and reducing hospital/ clinic visits [15].

The OCTOVA trial is the first randomised trial to evaluate O and the combination of O + C in women who have also had a prior PARPi and to evaluate response based on *BRCA1/2* mutational status. Within the trial, 30% of patients had known deleterious germline *BRCA1/2* mutations and 90% of study population had primary or acquired platinum-resistant and refractory ovarian cancer. The demographics and baseline characteristics of our study population are reflective of real-world patients who have limited lines of subsequent therapy available to them.

There was no evidence of a difference between O and wP (HR = 0.89, 60% CI: 0.72, 1.09; *P* = 0.69). Subgroup analysis suggests that O could be considered instead of wP in patients with a *BRCA1/2* mutation, particularly as it allows oral rather than weekly IV administration, a more acceptable side effect profile and no difference in quality of life. The numbers were too small to perform a separate analysis to assess PFS in participants who had received prior PARP inhibitor therapy. This is an important consideration as the first results from the Phase 3 OREO trial have demonstrated that, although retreatment with olaparib following prior exposure can be considered in women with platinum-sensitive relapse, the absolute magnitude of benefit was small (median improvement in PFS of 1.5 mo) [16].

Previous trials that have evaluated PARP inhibitors in women with relapsed platinum-resistant disease have also demonstrated that activity is related to *BRCA1/2* mutational status, with response rates of 30% with olaparib in patients with a *BRCA1/2* mutation [7] compared to trials such as Quadra (niraparib) [8] and CLIO (olaparib) [17] where response rates have been 3–13%, respectively (median PFS < 3 months) in patients who are *BRCA1/2* wild-type.

In *BRCA1/2*-mutated patients, the ARIEL4 trial compared rucaparib treatment to chemotherapy in both platinum-sensitive and -resistant disease relapse and demonstrated a median progression-free survival of 7.4 months (95% CI 6.7–7.9) in the rucaparib group versus 5.7 months (5.5–6.7) in the chemotherapy group (HR 0·67 [95% CI 0.52–0.86]; *P* = 0.0017) with objective response rates of 38% to rucaparib in the overall population compared to 32% (*P* = 0.13) with chemotherapy. In the platinum-resistant group the median progression-free survival with rucaparib was 6.4 (5.5–7.4) months vs 5.7 (3.7 vs 7.3) months with chemotherapy (HR 0.78 [95% CI 0.54–1.13]; *P* = 0.19).

Olaparib and rucaparib were until recently approved in platinum-resistant disease in the US. However, the manufacturers have withdrawn the treatment indication for olaparib, rucaparib and niraparib in heavily pre-treated BRCA mutated patients. Updated unplanned subgroup analysis from the ARIEL4 trials showed a potentially detrimental effect on survival in patients treated with rucaparib. In addition, patients who had received at least two lines of therapy in the SOLO3 trial, 65.2% of those who received olaparib died by the April 2021 data cut-off compared with 52.3% of those assigned to chemotherapy. For patients who received 3 or more prior lines of chemotherapy, 70.0% of patients in the olaparib arm had died compared with 54.8% of those assigned to chemotherapy (important prescribing information: Lynparza. AstraZeneca. August 26, 2022. Accessed September 22, 2022. https://bit.ly/3C4k4ud).

The OCTOVA trial has demonstrated that the combination of olaparib and cediranib has promising activity in platinum-resistant patients with an improved median PFS (5.4 months) compared to olaparib (3.7 months) alone irrespective of BRCA status, prior PARPi exposure or prior anti-angiogenic therapy (HR = 0.73; 60% CI: 0.59, 0.89; *P* = 0.1). This oral combination could therefore represent a potential alternative approach in this setting, particularly as it did not negatively impact quality of life compared to single-agent therapy with weekly paclitaxel (or olaparib).

The results of the OCTOVA trial add to the body of data regarding the combination of olaparib and cediranib in platinum-resistant disease from other recent Phase I–II trials such as BAROCCO [18] and CONCERTO [19]. The BAROCCO trial evaluated the combination of olaparib and either continuous or intermittent cediranib in patients with platinum-resistant disease. The PFS for the paclitaxel, the continuous, and the intermittent schedules were 3.1, 5.6 and 3.8 months; with the HR for PFS in the continuous arm vs control 0.76 (90% CI: 0.50–1.14, *P* = 0.265). Interestingly the intermittent treatment arm was shown to be inferior with the HR for PFS in the intermittent arm vs control 1.03 (90% CI: 0.68–1.55, *P* = 0.904) [18]. The results with olaparib and continuous cediranib were similar to the efficacy seen in the OCTOVA trial, although none of the BAROCCO patients had received prior PARP inhibitor therapy.

The Phase 1 CONCERTO trial, presented at ASCO 2020, evaluated olaparib and cediranib in 60 women with multiply treated (>3 prior lines of therapy) platinum-resistant disease who were *BRCA1/2* wild-type [19]. None had received prior PARP inhibitors, but the majority had received prior anti-angiogenic therapy (53/60). The overall response rate to olaparib and cediranib was 15.3% and median PFS was 5.1 months and median OS 13.2 months. The efficacy data from this trial were in keeping with the response rates and median progression-free survival and toxicity seen in the OCTOVA trial.

The EVOLVE trial, a multicentre single-arm Phase II trial, evaluated the addition of cediranib at the point of PARP inhibitor resistance in 34 heavily pre-treated patients with relapsed ovarian cancer. This study demonstrated that the activity of olaparib and cediranib combination varied according to the PARPi resistance mechanism. Objective responses were seen in 3/34 patients and patients with reversion mutations in homologous recombination genes and/or *ABCB1* upregulation had the poorest outcomes [20].

In conclusion, patients with relapsed ovarian cancer, and particularly those who have platinum-resistant disease, have limited therapeutic options. In this setting the control of symptoms remains of value, but reducing the burden of frequent treatment visits and toxicities such as alopecia are key aims. The OCTOVA trial results demonstrate that the combination of olaparib and cediranib may be a new therapeutic option for women with platinum-resistant disease regardless of their *BRCA1/2* mutational status and prior exposure to a PARP inhibitor or anti-angiogenic therapy. This is of importance as olaparib and bevacizumab are now increasingly used in the frontline setting further reducing treatment options in platinum-resistant disease. We await the results of the Phase III NCT02502266/NRG GY005 trial to determine if this combination may become a new potential treatment option for women with relapsed platinum-resistant ovarian cancer.

### Study limitations

OCTOVA is a Phase II trial with relatively small numbers of patients in each of the study arm. A larger Phase III evaluation of O + C vs O or C alone or chemotherapy is ongoing (NCT02502266/NRG GY005), and the results will determine the value of this combination in patients with platinum-resistant OC.

OCTOVA had to be amended due to slow recruitment of women with *BRCA1/2* mutation, as PARP inhibitors became the standard of care. The trial was amended after 27 patients had been recruited to include *BRCA1/2* wild-type patients. We also extended the platinum-free interval to include patients who had relapsed within 12 months of prior platinum due to concerns about the lack of activity of single agent olaparib in the platinum-resistant *BRCA1/2* wild-type population. The impact of this was minimised by adding *BRCA1/2* mutational status as an additional stratification factor to ensure that the three treatment arms remained balanced. Despite these changes, the majority of women treated on the OCTOVA trial (90%) had relapsed within 6 months of previous platinum and were defined as having platinum-resistant disease. There were however some unavoidable imbalances in the patient groups, 63% of the wP compared to 60% and 55% of the O and O + C groups, respectively, had two or fewer lines of therapy, and the BRCA1/2 mutation rate was also lowest in the wP group (35%), and this may therefore represent patients with more treatment-resistant biology.

The primary endpoint was RECIST-defined progression or death. The additional 13/139 (9.4%) who had non-RECIST-defined progression (e.g. clinical progression) were not included in the primary analysis. A separate analysis was conducted, including these participants and the results were in line with the main trial analysis.

OCTOVA was not developed to compare wP with O + C and a formal health economic assessment has not been performed of the additional costs of oral combination therapy compared to wP.

The AURELIA trial demonstrated that the addition of bevacizumab to weekly paclitaxel led to a significant improvement in PFS in platinum-resistant patients (HR = 0.48, 95% CI: 0.38–0.60): the median PFS was 3.4 months with chemotherapy versus 6.7 months with the combined regimen [21] However, bevacizumab for platinum-resistant disease has not been uniformly approved and is not the standard of care in all countries, including the UK. This is however a potential limitation of the activity of the wP arm within the OCTOVA trial.

Further biomarker analysis to determine the tumour HRD status (including BRCA) on archival samples is planned to determine if this had an impact on response to treatment, as the interaction between HRD status and combined anti-angiogenic and PARPi is unclear.

Recent analysis from the GY0004 trial, presented at ESMO 2021, did not demonstrate a predictive correlation between HR status and response to olaparib and cediranib [22]. TIE2 has been shown to be predictive of a response to anti-angiogenic agents in biliary and colorectal cancer [23] and is currently being evaluated in the frontline setting in ovarian cancer in the VALTIVE 1 trial (NCT 04523116). There is further scope to evaluate the changes in TIE2 in samples from the OCTOVA trial and assess if there is a predictive role for TIE2 as a biomarker for cediranib activity.

## Data Availability

The data collected for the study, including individual participant data and a data dictionary defining each field in the set, will be made available to researchers on request to the study team and with appropriate reason when accompanied by a peer-reviewed protocol, with publication and on agreement of the Independent Early Phase Trials Oversight Committee (IEPTOC). The shared data will be deidentified participant data, and will be available for 5 years following publication of the study. Data will be shared with investigator support, after approval of a proposal, with a signed data access agreement.
